# Applying Acylated Fucose Analogues to Metabolic Glycoengineering

**DOI:** 10.3390/bioengineering2040213

**Published:** 2015-11-30

**Authors:** Julia Rosenlöcher, Verena Böhrsch, Michael Sacharjat, Véronique Blanchard, Christoph Giese, Volker Sandig, Christian P. R. Hackenberger, Stephan Hinderlich

**Affiliations:** 1 Laboratory of Biochemistry, Department of Life Sciences and Technology, Beuth University of Applied Sciences Berlin, Seestrasse 64, 13347 Berlin, Germany; E-Mail: rosenloecher@beuth-hochschule.de; 2 Department of Biology, Chemistry and Pharmacy, Freie Universität Berlin, Takustrasse 3, 14195 Berlin, Germany; 3 Leibniz-Institut für Molekulare Pharmakologie (FMP), Robert-Rössle-Strasse 10, 13125 Berlin, Germany; E-Mails: verena.boehrsch@alumni.tu-berlin.de (V.B.); hackenbe@fmp-berlin.de (C.P.R.H.); 4 ProBioGen AG, Goethestrasse 54, 13086 Berlin, Germany; E-Mails: michael.sacharjat@probiogen.de (M.S.); christoph.giese@probiogen.de (C.G.); volker.sandig@probiogen.de (V.S.); 5 Institute of Laboratory Medicine, Clinical Chemistry and Pathobiochemistry, Charité-Universitätsmedizin Berlin, Augustenburger Platz 1, 13353 Berlin, Germany; E-Mail: veronique.blanchard@charite.de; 6 Department of Chemistry, Humboldt-Universität zu Berlin, Brook-Taylor-Strasse 2, 12489 Berlin, Germany

**Keywords:** acylation, fucose analogues, fucosylation, glycosylation, metabolic glycoengineering, salvage pathway

## Abstract

Manipulations of cell surface glycosylation or glycan decoration of selected proteins hold immense potential for exploring structure-activity relations or increasing glycoprotein quality. Metabolic glycoengineering describes the strategy where exogenously supplied sugar analogues intercept biosynthetic pathways and are incorporated into glycoconjugates. Low membrane permeability, which so far limited the large-scale adaption of this technology, can be addressed by the introduction of acylated monosaccharides. In this work, we investigated tetra-*O*-acetylated, -propanoylated and -polyethylene glycol (PEG)ylated fucoses. Concentrations of up to 500 µM had no substantial effects on viability and recombinant glycoprotein production of human embryonic kidney (HEK)-293T cells. Analogues applied to an engineered Chinese hamster ovary (CHO) cell line with blocked fucose *de novo* synthesis revealed an increase in cell surface and recombinant antibody fucosylation as proved by lectin blotting, mass spectrometry and monosaccharide analysis. Significant fucose incorporation was achieved for tetra-*O*-acetylated and -propanoylated fucoses already at 20 µM. Sequential fucosylation of the recombinant glycoprotein, achieved by the application of increasing concentrations of PEGylated fucose up to 70 µM, correlated with a reduced antibody’s binding activity in a Fcγ receptor IIIa (FcγRIIIa) binding assay. Our results provide further insights to modulate fucosylation by exploiting the salvage pathway via metabolic glycoengineering.

## 1. Introduction

Glycosylation is a highly versatile, complex and ubiquitous form of post-translational modification. Glycans, covalently attached carbohydrate moieties, participate in a variety of intracellular processes or dictate basic protein characteristics such as biological activity, protein folding or proteolytic stability [1]. Moreover, glycans decorate eukaryotic cell surfaces mediating multiple binding and recognition events of cell-matrix and cell-cell communication [2].

The biosynthesis of glycans is non-template driven and is rather realized by a complex and sequential interaction of enzymes (glycosyltransferases and glycosidases). Manipulations and studies of glycosylation cannot be achieved by conventional genetic approaches. In this regard, metabolic glycoengineering, also referred to as metabolic oligosaccharide engineering, is a straightforward and nascent strategy to create modified glycans by incorporation of exogenously supplied sugar analogues. A landmark work was published in 1992 by Reutter and coworkers [3], which reported on the substrate permissivity of the sialic acid biosynthesis pathway for derivatives of the metabolic precursor *N-*acetylmannosamine (ManNAc) with unnatural elongations at the *N-*acyl position. Subsequently, a multitude of functional groups (such as azides, thiols, alkynes or ketones) were introduced and served as targets for chemoselective ligation reactions enabling glycan detection for quantitative and functional studies or visualization and localization of glycoconjugates [4]. Since the sialic acid metabolism served as a workhorse for methodology development and pioneered this technique [5], the scope of metabolic glycoengineering has been broadened to further glycosylation pathways. In this regard, the deoxyhexose fucose was addressed as the first non-amino sugar. Owing to their terminal position at glycan structures and their relevance in important cellular and pathological processes such as fertilization, immune response, inflammation or tumor metastasis [6], the study of fucosylated glycoconjugates is of high interest. Structural modifications of the fucose C-6 position by azide or alkyne substituents were shown to be tolerated by fucosyltransferases and the biosynthetic pathway in several reports of the Bertozzi and Wong group, respectively [7,8,9].

Besides visualizing the dynamics and localization of glycans on cell surface level or imaging their intracellular distribution via introduced structural modifications, also unmodified sugars and metabolic precursors are a key aspect of ongoing research. On the level of cell surface glycoconjugates, uridine diphosphate-*N‑*acetylglucosamine (GlcNAc) 2-epimerase (part of the bifunctional enzyme GNE [10]) was reported to catalyze the rate-limiting step in the biosynthetic pathway of sialic acids and, thus, be a major determinant of cell surface sialylation. In this context, hyposialylation was shown to be metabolically complemented by ManNAc treatment in human hematopoietic cell lines [11]. Monosaccharide therapy of GNE myopathy, a neuromuscular genetic disorder associated with impaired glycan sialylation, was investigated by oral administration of mannosamine (ManN) and ManNAc [12] or sialic acid [13] in mouse models, respectively. Moreover, oral mannose is an efficient therapy for patients of phosphomannose isomerase deficiency—the only congenital defect of glycosylation that can be treated [14]. Experiments based on findings of Marquardt and colleagues revealed that oral supplementation of fucose and incorporation via salvage pathway correct the metabolic defects found in patients with leucocyte adhesion deficiency [15]. In a biotechnological approach, Gu and Wang addressed the sialylation level of recombinant human interferon-γ by supplementing the CHO culture medium with ManNAc [16]. In a similar study, Bork *et al.* increased the sialylation of recombinant erythropoietin by ManNAc treatment [17]. Further potentials of metabolic glycoengineering for biotechnological applications were also reported for recombinant sialo-glycoproteins expressed in insect cells [18].

Large-scale adaption of metabolic glycoengineering on a cellular level is hindered by low metabolic utilization of sugars or their analogues requiring millimolar quantities of the monosaccharides to overcome plasma membrane barrier and maximize efficient incorporation [4]. Masking the hydroxyl groups of sugar residues by acetylation (that is removed intracellularly by unspecific esterases) was introduced by Sarkar *et al.* in 1995 as a strategy to increase hydrophobicity and cellular uptake [19]. Acetylated ManNAc derivatives were later reported to be used with up to 900-fold improved efficiency in contrast to their underivatized counterparts in various mammalian cell lines [20]. Further elongations of the carbon chain length of ester derivatives attached to the hydroxyl groups (as for tetra-*O*-propanoylated or -butanoylated ManNAc) were also cleaved by cytosolic esterases and resulted in additional increase of metabolic utilization at low concentrations (<150 µM), but also showed enhanced toxicity at higher concentrations (such as 500 µM) [21].

**Figure 1:**
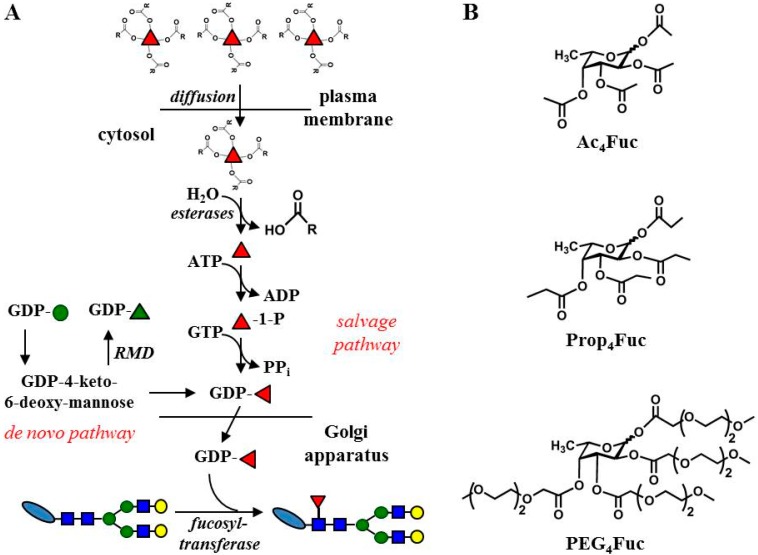
Schematic outline of the experimental approach used in this study (**A**). Biosynthesis of L-fucose (red triangle) occurs mainly via *de novo* synthesis and, to a minor extent, through the salvage pathway that is exploited here in the course of metabolic glycoengineering; Acyl moieties (shown in **B**) are introduced to fucose to facilitate cellular uptake across the plasma membrane and are thought to be cleaved intracellularly by cytosolic esterases. Emanating from GDP-mannose (green circle), the *de novo* pathway can be blocked by enzymatic conversion of the intermediate GDP-4-keto-6-deoxymannose into a dead-end product (GDP-rhamnose, green triangle) by GDP-6-deoxy-D-lyxo-4-hexulose reductase (RMD) as previously shown in [22]. Other monosaccharide symbols are explained in Section 2.8.

The current work addresses the fucose salvage pathway via the metabolic glycoengineering method. According to the approach of Kim *et al.* [21], we transferred the basic principle from ManNAc to fucose applying acylated analogues to facilitate passive diffusion across the cell membrane and, thus, cellular uptake (Figure 1). A tetra-*O*-propanoylated derivative (Prop_4_Fuc) was used in comparison to standard acetylated fucose (Ac_4_Fuc). Since the elongation of ester groups can potentially raise solubility or toxicity problems, we also investigated the usage of a novel PEGylated fucose (PEG_4_Fuc). Due to the activity of cytosolic esterases with broad substrate spectra and the absence of any modifying group, we assumed that our analogues are introduced in the form of unmodified fucose units into the biosynthesis pathway (Figure 1). Thus, a proof of successful monosaccharide incorporation had to be provided by an increase of fucosylation itself. As an appropriate test system in order to clearly distinguish the effects of exogenously supplied sugar, we chose a CHO cell line in which the *de novo* fucose synthesis pathway is blocked by heterologous expression of the prokaryotic enzyme GDP-6-deoxy-D-lyxo-4-hexulose reductase (RMD) (GlymaxX^®^ technology [22], see Figure 1). We also focused on a recombinantly expressed and secreted immunoglobulin G (IgG) antibody in these CHO cells. Fucosylation is known to crucially affect one of the antibody’s effector functions: antibodies lacking core fucose in their Fc *N-*glycan exhibit significantly increased antibody-dependent cellular toxicity (ADCC) [23]. In this regard, we aimed at a sequential antibody fucosylation by applying controlled concentrations of fucose analogues in our metabolic glycoengineering experiments. Moreover, the relation between the fucosylation level and its effect on IgG activity were investigated via a FcγRIIIa binding assay indicating ADCC bioactivity.

## 2. Experimental Section

### 2.1. Materials

Dulbecco’s modified eagle medium (DMEM) with 4.5 g/L glucose, L-glutamine, penicillin/streptomycin and Dulbecco’s phosphate buffered saline (DPBS) were obtained from PAN-Biotech GmbH (Aidenbach, Germany), adenovirus expression medium (AEM) from Life Technologies GmbH (Darmstadt, Germany), fetal calf serum (FCS) superior from Merck Millipore (Darmstadt, Germany) and L-fucose from Sigma-Aldrich GmbH (Taufkirchen, Germany). Unless otherwise stated, all chemicals were purchased from Carl Roth GmbH + Co. KG (Karlsruhe, Germany).

### 2.2. Syntheses of Fucose Analogues

Fucose derivatives were prepared according to standard procedures with non-optimized syntheses. Details are given in the Supplementary Information. Therefore, pyridine and triethylamine were dried and stored over potassium hydroxide. Technical grade solvents were distilled prior to use. Column chromatography was performed on silica gel 60 (Merck Millipore, Darmstadt, Germany). Thin-layer chromatography (TLC) was run on Merck classical TLC silica gel 60 F_254_ plates and evaluated in UV light (254 nm) subsequent to staining with anisaldehyde/sulfuric acid in ethanol. ^1^H-nuclear magnetic resonance (NMR) and ^13^C-NMR spectra were recorded on a JEOL ECX-400 (JEOL GmbH, Freising, Germany) or a Bruker Avance 300 spectrometer (Bruker BioSpin GmbH, Rheinstetten, Germany) in CDCl_3_ (Deutero GmbH, Kastellaun, Germany). Chemical shifts were determined relatively to tetramethylsilane using the residual solvent peak as internal calibration signal. Infrared (IR) spectra were measured on a Nexus FT-IR spectrometer (former Thermo Electron Cooperation, Madison, WI, USA) equipped with a Nicolet Smart DuraSampIIR ATR. Masses were analyzed on an Agilent 6210 ESI-TOF (Agilent Technologies, Santa Clara, CA, USA) using a solvent flow rate of 4 µL/min , spray voltage of 4 kV and the desolvation gas set to 15 psi (1 bar). All other parameters were optimized for maximal abundance of [M+H_2_O]^+^ or [M+Na]^+^ ions. Anomeric mixtures of the products were used.

### 2.3. Cell Culture

Standard cultivation of all cells was performed in medium supplemented with 2 mM L-glutamine and penicillin/streptomycin (100 U/mL, 100 µg/mL) within a 5% CO_2_ atmosphere at 37 °C. Stably alpha-1-antitrypsin (A1AT)-expressing HEK-293T cells [24] were cultivated under serum-containing conditions in DMEM complemented with 10% (v/v) FCS or in serum-free AEM. Parental CHO cells and the CHO RMD cell line—each engineered for overexpression and secretion of a biosimilar version of IgG1-type therapeutic antibody trastuzumab (Herceptin^®^) [22]—were grown in suspension and maintained in cell culture flasks in serum-free AEM.

### 2.4. Cell Treatment with Acylated Fucose Analogues

100 mM stock solutions of the synthesized fucose analogues were prepared either in DMSO (Ac_4_Fuc and Prop_4_Fuc) or sterilized water (PEG_4_Fuc). HEK-293T A1AT cells were washed in DPBS prior to seeding in 96-well plates at a density of 4.5 × 10^3^ cells/well (DMEM) or 9 × 10^3^ cells/well (AEM) and a subsequent incubation of 24 h. CHO cells were prepared likewise, but seeded in AEM at a density of 1.8 × 10^5^ cells/mL in T75 culture flasks. Afterwards, the fucose analogue dilutions were added to the cultivated cells to give final concentrations ranging between 0 and 500 µM followed by a three-day incubation of the treated cells. HEK-293T cells were referred to cell viability check while the supernatant was analyzed for recombinant A1AT expression. CHO cells were harvested by centrifugation at 150 × *g* for 5 min and the pellet was washed thoroughly by DPBS twice prior to membrane protein isolation. CHO cell supernatants (containing the recombinant antibody trastuzumab) were subjected to subsequent affinity purification.

### 2.5. Measurement of Cell Viability and Impact on Recombinant Protein Expression

Cell viability was determined in quadruplicates within a 96-well plate format by metabolic activity of mitochondrial enzymes using the colorimetric 3-(4,5-dimethylthiazol-2-yl)-2,5-diphenyltetrazolium bromide (MTT) assay based on a protocol of Mosmann (1983) [25] with minor modifications. In brief, 10 µL of a 5 mg/mL stock solution of MTT in DPBS were added to prepared cells and subsequently plates were incubated for 2 h at 37 °C. The crystals of the purple formazan product could be dissolved using 100 µL of MTT solvent (4 mM HCl, 0.1% (v/v) Nonidet P-40 in isopropanol) followed by incubation on an orbital shaker for 20 min at room temperature. Absorbance was read spectrophotometrically at 595 nm and a reference wavelength of 630 nm. Relative cell viability was calculated by relating the absorbance of treated cells to untreated controls.

Within a parallel and independent approach, the effects of exogenously supplied fucose analogues on recombinant glycoprotein production were analyzed via reducing sodium dodecyl sulfate polyacrylamide gel electrophoresis (SDS-PAGE) with subsequent electroblotting and immunostaining. Aliquots of HEK-293T cell supernatants were taken from the incubated 96-well plates and centrifuged at 150 × *g* for 5 min to remove cells and debris prior referring them to gel electrophoresis. Chemiluminescence detection used a monoclonal horseradish peroxidase-coupled antibody to human A1AT from sheep (The Binding Site GmbH, Schwetzingen, Germany). In terms of trastuzumab production in CHO RMD cells, same sample volumes of simultaneously purified IgG (see Section 2.9) were referred to SDS-PAGE with subsequent Bio-Safe™ Coomassie (Bio-Rad Laboratories GmbH, Munich, Germany) staining.

### 2.6. Isolation of Membrane Proteins

The isolation protocol of CHO membrane proteins followed a previously published procedure [26] with minor modifications. About 4 × 10^7^ cells were used for cell lysis. After dissolving the membrane protein-rich cell pellet in a detergent-containing phosphate buffer, an incubation period of 2 h and periodic shaking at room temperature was implemented to maximize the protein resuspension process. Subsequent to detergent removal, samples were directly assayed for protein concentration using Pierce™ BCA Protein Assay Reagent kit (Life Technologies GmbH, Darmstadt, Germany).

### 2.7. Release and Separation of N-Linked Glycans

Appropriate samples contained 40 µg of purified trastuzumab or 250 µg of isolated membrane proteins. The latter underwent boiling for 5 min prior to enzyme treatment. Tryptic digestion was performed for 4 h at 37 °C using 2.5 µg trypsin (Sigma-Aldrich GmbH, Taufkirchen, Germany) per 30 µg of glycoprotein. Subsequent to enzyme deactivation (5 min, 95 °C), a second trypsin addition and overnight incubation at 37 °C was carried out. Releasing *N*-linked glycans, samples were incubated for 5 min at 95 °C and treated with 0.5 U (in case of the purified antibody) or rather 1.5 U (for isolated membrane proteins) of *N-*glycosidase F from *Flavobacterium meningospeticum* (Roche Diagnostics GmbH, Mannheim, Germany). After 4 h of incubation at 37 °C, the enzyme addition was repeated, accomplished overnight and followed by *N*-glycosidase F inactivation (5 min, 95 °C). *N-*glycans were separated from the peptide fraction using reversed-phase C18 cartridges and a subsequent desalting step on graphitized cartridges (Grace Davison Discovery Sciences, Worms, Germany) as described before [27]. Purified *N-*glycans were lyophilized and stored, if applicable, at −20 °C.

### 2.8. Permethylation and Matrix-Assisted Laser Desorption/Ionization Time-of-Flight (MALDI-TOF) Mass Spectrometry

Since permethylation converts the sialic acid residues of glycans to methyl esters, these terminal monosaccharides are stabilized while improving the efficiency of positive ion formation [28]. The derivatization procedure followed standard protocols of the solid sodium hydroxide technique [28,29] with minor modifications. In this regard, all incubations were carried out under conditions of continuous shaking at room temperature. The iodomethane reaction was stopped by the addition of chloroform and subsequent washing steps with water until achieving a neutral pH of the aqueous phase. Dried *N*-glycans were dissolved in 75% (v/v) acetonitrile in water and mixed with super-dihydroxybenzoic acid (sDHB) matrix (Sigma-Aldrich GmbH, Taufkirchen, Germany). Recording of mass spectra on an Ultraflex III MALDI-TOF/TOF spectrometer (Bruker Daltonik GmbH, Bremen, Germany) and subsequent data processing was realized as reported previously [30]. Evaluations considered all detected *N-*glycan signals with relative peak areas higher than 0.5%. Schematic representation of glycan structures based on the symbol nomenclature of the Consortium for Functional Glycomics [31]: green circle, mannose; yellow circle, galactose; blue square, GlcNAc; yellow square, *N-*acetylgalactosamine (GalNAc); white square, *N-*acetylhexosamine (HexNAc); red triangle, fucose; purple diamond, *N-*acetylneuraminic acid. Monosaccharide linkage was not further investigated by fragmentation analyses via tandem mass spectrometry or exoglycosidase digestions. Therefore, the simplified representation of the glycan structures comprised brackets if more than one potential position of the respective sugar should be pointed out.

### 2.9. Trastuzumab Purification

Recombinant protein production was proved via reducing SDS-PAGE and subsequent Bio-Safe™ Coomassie (Bio-Rad Laboratories GmbH, Munich, Germany) staining of collected CHO cell culture supernatants. Affinity purification of trastuzumab was performed by the use of protein G agarose (Roche Diagnostics GmbH, Mannheim, Germany) according to the manufacturer’s instructions. The antibody was eluted with 50 mM glycine pH 2.5. Integrity and purity was confirmed by reducing SDS-PAGE and Coomassie staining. Samples underwent dialysis against 1 mM NaH_2_PO_4_, 5 mM NaCl and 1 mM EDTA (pH 7.5) followed by a determination of protein concentration using the Pierce™ BCA Protein Assay Reagent kit already mentioned above.

### 2.10. Lectin Blotting

Protein samples of 1–2 µg were separated by reducing SDS-PAGE and transferred to a nitrocellulose membrane via standard electroblotting. The membrane was blocked for at least 1 h at room temperature (or at 4 °C overnight) in PBS containing 0.05% (v/v) Tween-20 (PBS-T) with 5% (w/v) bovine serum albumin (BSA). Membrane rinsing was followed by an incubation with a 1 µg/mL solution of biotinylated *Aleuria aurantia* lectin (AAL-I) (Vector Laboratories, Burlingame, CA, USA) in PBS-T for 1.5 h. Membranes were thoroughly washed with PBS-T and incubated with a 0.5 µg/mL streptavidin-peroxidase conjugate (Sigma-Aldrich GmbH, Taufkirchen, Germany) in PBS-T containing 3% (w/v) BSA for 1 h. Subsequent to membrane washing with PBS, chemiluminescence detection was carried out and documented using the Image Reader LAS-4000 system (Fujifilm Europe GmbH, Düsseldorf, Germany).

### 2.11. High-pH Anion-Exchange Chromatography with Pulsed Amperometric Detection (HPAEC-PAD) Monosaccharide Analysis

Respectively, 20 µg of purified trastuzumab or 30 µg of isolated CHO membrane proteins were hydrolyzed in quadruplicates in 4 M trifluoroacetic acid (TFA) for 4 h at 100 °C. Human alpha-1 acid glycoprotein (former Boehringer Mannheim GmbH, Mannheim, Germany) and a blank sample were used as positive and negative controls, respectively. As internal standards 2-deoxyribose, fructose and melibiose (each from Sigma-Aldrich GmbH, Taufkirchen, Germany) were used. HPAEC-PAD was performed on an ICS-3000 Ion Chromatography System (Dionex GmbH, Dreieich, Germany) using a Dionex CarboPac^®^ PA200 column. Neutral monosaccharides were separated by isocratic 2.25 mM NaOH elution while post-column addition of 200 mM NaOH provided the conditions for amperometric detection. Data represent the mean of four sample preparations and variation coefficients were below 10% for all experiments.

### 2.12. FcγRIIIa Binding Assay

In order to determine the binding activity of purified trastuzumab samples to the ADCC-relevant FcγRIIIa, the assay was conducted as previously described [22]. In brief, receptor binding was detected by an enzyme-linked immunosorbent assay using the recombinant histidine-tagged FcγRIIIa molecule (R&D Systems, Wiesbaden, Germany) as a capture reagent, an anti-human IgG peroxidase-conjugated antibody for detection (Dianova GmbH, Hamburg, Germany) and tetramethylbenzidine (Seramun Diagnostica GmbH, Heidesee, Germany) as chromogenic substrate. Based on the data of concentration-dependent absorption at 450 nm, median effective concentration (EC50) values were determined by a four-parameter sigmoid curve model using Origin 8.1 software (Additive GmbH, Friedrichsdorf, Germany).

## 3. Results and Discussion

### 3.1. Effects of Fucose Analogues on HEK-293T Cell Viability and Recombinant A1AT Expression

**Figure 2:**
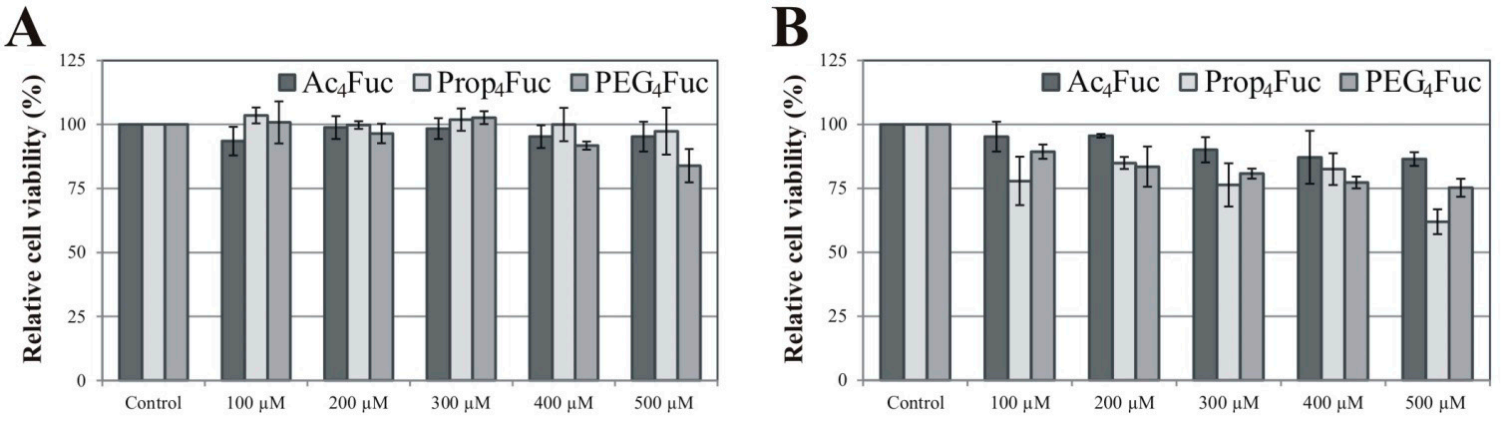
Viability of recombinant HEK-293T/A1AT cells measured by MTT assay subsequent to a treatment with acylated fucose analogues for three days. Culture conditions in serum-containing DMEM (**A**) are opposed to serum-free AEM (**B**). Data represent the mean ± standard deviation of quadruplicates and are related to an untreated control.

Considering acylated fucose analogues for application in metabolic glycoengineering approaches, we initially analyzed their cytotoxicity. HEK-293T cells were chosen as a primary test system since these cells are extensively used and considered as a standard in academic and industrial research. The option to shift between serum-supplemented and serum-free cultivation results in adherent (serum-supplementation) and suspension (serum-free) cell growth. Besides this strong influence on cellular behavior, serum supplementation with FCS is, above all, a rich source of glycoconjugates and free monosaccharides that compete with exogenously supplied derivatives. Analysis of HEK-293T cells under serum-containing and serum-free conditions therefore allows a comparative study including several important parameters. Viability of recombinant HEK-293T/A1AT cells was determined by MTT assay subsequent to a three-day incubation of concentrations up to 500 µM of Ac_4_Fuc, Prop_4_Fuc and PEG_4_Fuc, respectively (Figure 2).

Under serum-supplemented conditions (Figure 2A), reduction of HEK-293T cell viability was negligible for all fucose analogues up to 500 µM compared to the untreated control (0 µM). Outcomes in the absence of FCS (Figure 2B) were a little more apparent, but viability remained above 75% for all treatments up to 400 µM. No distinct tendencies of tolerance could be detected for a particular fucose modification. This is in contrast to the effects of the acylated equivalents of ManNAc previously investigated in experiments with Jurkat cells under serum-containing conditions [21]. These suspension-growing cells showed a viability reduction of more than 50% for propanoylated ManNAc (500 µM) while the effects of acetylated ManNAc were merely around 5%. For the Prop_4_ManNAc derivative, a careful control of cell density and analogues concentration [20] or—as an additional strategy—the establishment of analogue-resistant cell lines [32] are required to overcome these toxicity effects. In case of Ac_4_Fuc, Prop_4_Fuc and PEG_4_Fuc, however, concentrations up to 500 µM are applicable. The concentration range for optimal metabolic flux and the prevention of toxic side effects may be quite narrow, as reported by Sampathkumar and colleagues (2006) using the example of *O‑*butanoylated ManNAc [33]. The toxic effect of acylated analogues is mainly due to the release and accumulation of organic acids subsequent to esterase cleavage (see Figure 1A), lowering the intracellular pH of the cultivated cells [4]. It is therefore likely that HEK‑293T cells have a higher buffer capacity compared to Jurkat cells. Furthermore, cultivation of HEK-293T cells in the presence of FCS seems to increase this protective effect.

Besides cell viability, the impact of exogenously supplied monosaccharides on recombinant protein expression is of great interest, particularly in terms of biotechnological applications. As a general guideline for untested cell lines, Aich and Yarema recommended studying novel acetylated analogues over the range of 0 to ~250 µM since metabolic incorporation usually does not increase above these concentrations [4]. In order to investigate a potential influence we analyzed the recombinant expression of the A1AT glycoprotein in HEK-293T cells when treated with raising concentrations of fucose analogues. Western blot studies of HEK-293T cell supernatants revealed A1AT production comparable to the untreated control for all feeding approaches under serum-containing and serum-free conditions (see Figure S1). Regarding these findings, we decided to apply not more than 200 µM of Ac_4_Fuc, Prop_4_Fuc and PEG_4_Fuc for all subsequent experiments.

### 3.2. Incorporation of Fucose Analogues on CHO RMD Cell Membrane

Successful analogue utilization has to be proved by increasing fucosylation levels since the introduced acyl moieties are assumed to be removed intracellularly by unspecific esterases. Since *de novo* synthesis normally accounts for 90% of available activated fucose [34], this pathway has to be blocked in order to achieve prominent metabolic effects of the applied fucose analogues. For this purpose, we utilized the CHO cell line expressing RMD from *Pseudomonas aeruginosa* that converts a substrate intermediate into GDP-rhamnose depleting *de novo* GDP-fucose synthesis (Figure 1, [22]). Neither the parental CHO cell line nor CHO RMD cells showed altered growth or morphology when the acylated fucose analogues were applied to the cell culture medium. Since the amount of recombinant protein expression is the final read-out corresponding to cell viability, we initially analyzed the recombinant production of the antibody trastuzumab that is overexpressed by these cells (see Figure S2). Concentrations up to 140 µM (as used for subsequent CHO experiments within this work) had no crucial effect on CHO RMD antibody production and, thus, might not have critically influenced cell viability.

**Figure 3:**
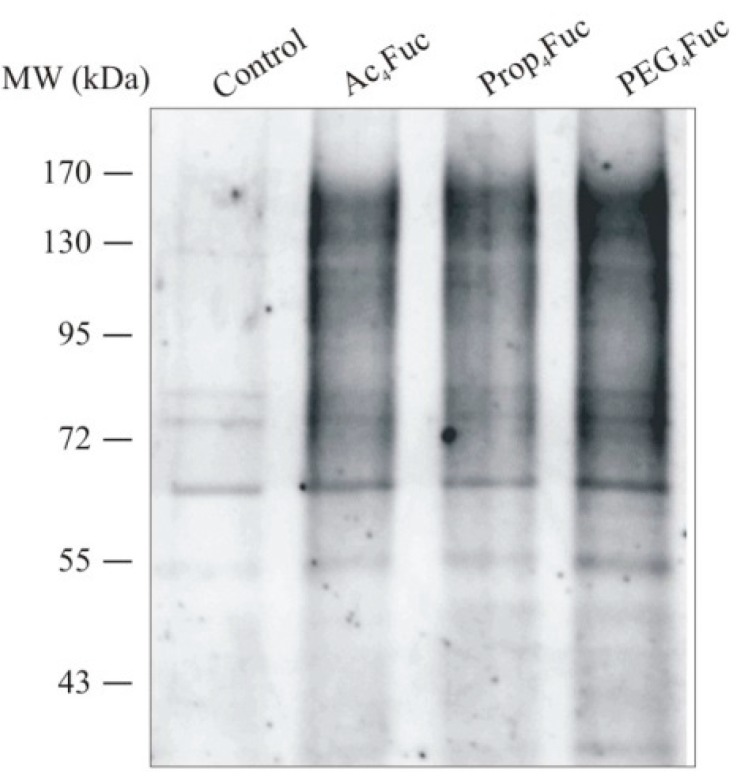
Lectin blot analysis of CHO RMD membrane proteins. Cells were treated with 80 µM of the respective fucose analogue under serum-free conditions for three days prior to an isolation of membrane proteins. 2 µg of the protein samples were separated by reducing SDS-PAGE, electroblotted and probed with AAL-I. An untreated CHO RMD sample served as control.

CHO RMD cells were treated with 80 µM of the respective monosaccharides under serum-free conditions followed by an isolation of membrane proteins from the collected cell pellets. A lectin blot survey using AAL-I with specificity to fucose in all binding positions (Figure 3) confirmed the potential to increase cell membrane fucosylation by all tested analogues.

**Figure 4:**
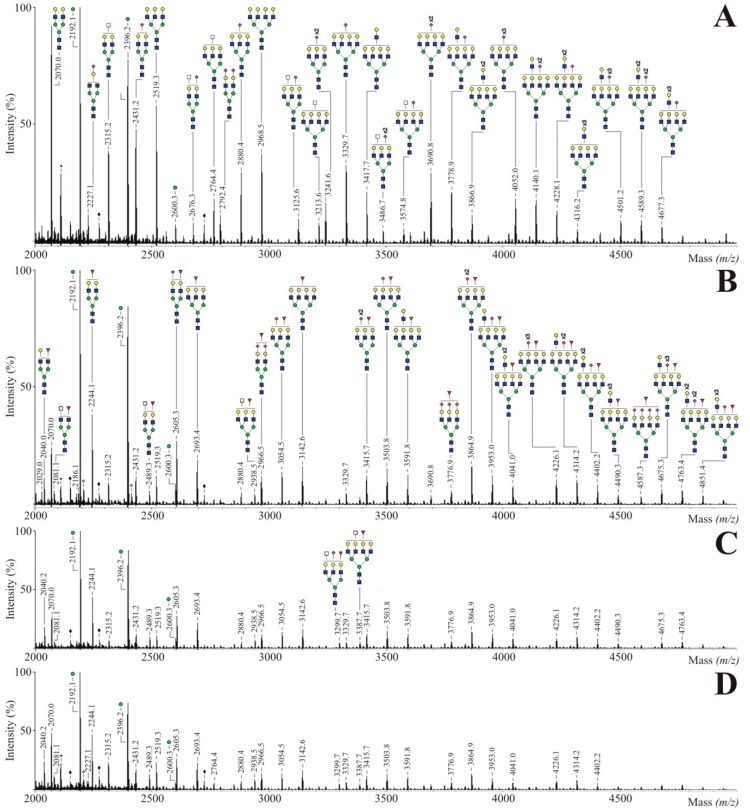
MALDI-TOF mass spectra of permethylated *N*-glycans of total CHO RMD cell membrane proteins after three-day treatment with 80 µM of Ac_4_Fuc (**B**), Prop_4_Fuc (**C**) or PEG_4_Fuc (**D**) under serum-free culture conditions; in comparison to an untreated control (**A**). Membrane-bound *N*-glycans were enzymatically released by *N*-glycosidase F. Molecular ions are present in either sodiated [M + Na]^+^ or potassiated [M + K]^+^ form (*). Highmannose *N-*glycans are marked by a green circle, polyhexose contaminations by a black ring (•) and unidentified peaks by a black diamond (♦). All signals with relative peak areas higher than 0.5% are labeled with their respective numerical *m/z*-values. Graphical visualization of all structures is only done once per *m/z* in the whole Figure (**A**–**D**).

In order to verify the utilization of Ac_4_Fuc, Prop_4_Fuc and PEG_4_Fuc during glycan synthesis, we investigated the *N-*glycan profiles of the isolated membrane fractions. 250 µg of CHO RMD proteins underwent tryptic digestion into glycopeptides followed by an enzymatic *N-*glycan release using *N-*glycosidase F. Samples were derivatized by permethylation and subsequently analyzed by MALDI-TOF mass spectrometry. Figure 4 displays the resulting spectra covering a mass range of *m/z* 2000–5000. As a consequence of the methodical conditions underlying the protein isolation procedure, signals of highmannose-type *N-*glycans (*m/z* 2192.1, 2396.2 and 2600.3) were detected in high portions. These prominent peaks are most likely caused—as already suggested by Reinke *et al.* [27]—by incompletely trimmed precursor oligosaccharide structures of the Golgi apparatus that cannot be separated from the plasma membrane fraction. Thus, data analysis within this work was rather focused on complex-type *N-*glycans.

Regarding the untreated control of CHO RMD membrane proteins (Figure 4A), a biantennary (*m/z* 2070.0), triantennary (*m/z* 2519.3) and tetraantennary *N-*glycan structure (*m/z* 2968.5) could be assigned to the main peaks. Structural diversity was characterized by varying sialylation levels or odd HexNAc molecules (which are not visualized in form of a bisecting GlcNAc branch since CHO cells lack the GlcNAc transferase III activity [35]). The occurrence of poly-*N*-acetyllactosamine (LacNAc) units, as a common structure of membrane glycoconjugates and a modulator of cell adhesion in matters of galectin binding [36], was also reported previously for CHO cells [37]. No fucosylated *N-*glycan structure was detected in the considered mass range confirming the defect of *de novo* fucose biosynthesis for CHO RMD [22]. Treatment of CHO RMD cells with fucose analogues (Figure 4B–D) resulted in *N-*glycans preferably bearing a fucose residue while a minor portion was assigned to afucosylated structures (e.g. *m/z* 2070.0, 2315.2 or 2880.4). Fucose was successfully incorporated into biantennary up to tetraantennary molecules with multiple LacNAc units. Mass spectra of Ac_4_Fuc (B), Prop_4_Fuc (C) and PEG_4_Fuc (D) showed comparable *N-*glycan profiles without substantial differences and merely negligible variations in peak intensities. Since signals with relative peak areas under 0.5% are unlabeled in Figure 4, the determination of this cut-off caused all apparent distinctions. As accepted for acetylated monosaccharides or shown for propanoylated ManNAc [21], all acyl moieties of the fucose analogues were removed prior to the incorporation into glycan structures, confirming the presence of unspecific esterases also active on the novel PEGylated fucose.

Mass spectrometry analysis of CHO cell membrane *N*-glycans resulted in semi-quantitative data only. We therefore performed quantitative monosaccharide analysis by HPAEC-PAD (see Figure S3). 30 µg of isolated CHO RMD membrane proteins underwent strong acid hydrolysis prior to monosaccharide separation by anion exchange chromatography. Data confirmed the absence of fucose for glycans of the untreated CHO RMD sample. No quantitative differences could be detected for the incorporation of Ac_4_Fuc, Prop_4_Fuc or PEG_4_Fuc on the level of CHO RMD membrane proteins. For all metabolic glycoengineering approaches molar ratios of fucose were in the range of 0.3 to three mannoses. The occurrence of GalN(Ac) is most likely due to the presence of *O*-glycans on CHO cells [37].

### 3.3. Incorporation of Fucose Analogues on Recombinant Trastuzumab Expressed in CHO Cells

Since the application of metabolic glycoengineering approaches is also of high interest and relevance for the biotechnological industry, utilization of acylated fucoses should also be investigated on recombinant protein level. IgGs show a low-complex and well described glycosylation profile (characterized by predominant complex-type biantennary *N-*glycans [38]) and fucosylation is of fundamental importance for one of the antibody’s effector functions (see introduction). We focused the IgG1-type trastuzumab, an antibody directed against the HER2 antigen on breast and gastric cancer cells [39], since FcγRIIIa binding and ADCC are critical for this antibody. In order to demonstrate the effects of RMD expression and to have a reference for natural CHO fucosylation of the antibody, we included the parental CHO cell line with functional fucose *de novo* synthesis in our study.

Within an initial proof of principle experiment, trastuzumab was isolated from both cell lines after four days of cultivation followed by an affinity purification using protein G agarose. Antibody purification out of collected cell supernatants and sample integrity is documented in Figure S4A,B and was comparable for both approaches. The fucosylation level of purified trastuzumab was investigated qualitatively by lectin blot analysis (Figure S4C). Data revealed no signal of fucose-specific AAL-I for the antibody derived from CHO RMD cells while a prominent band at around 50 kDa was apparent for trastuzumab generated with the parental CHO cell line. The detected signal agrees with the presence of the single *N-*glycan in trastuzumab linked to asparagine 297 of the constant heavy chain (CH2) domain [38]. The functional pathway including fucose biosynthesis and glycan fucosylation was confirmed for the CHO reference cell line that way.

**Figure 5:**
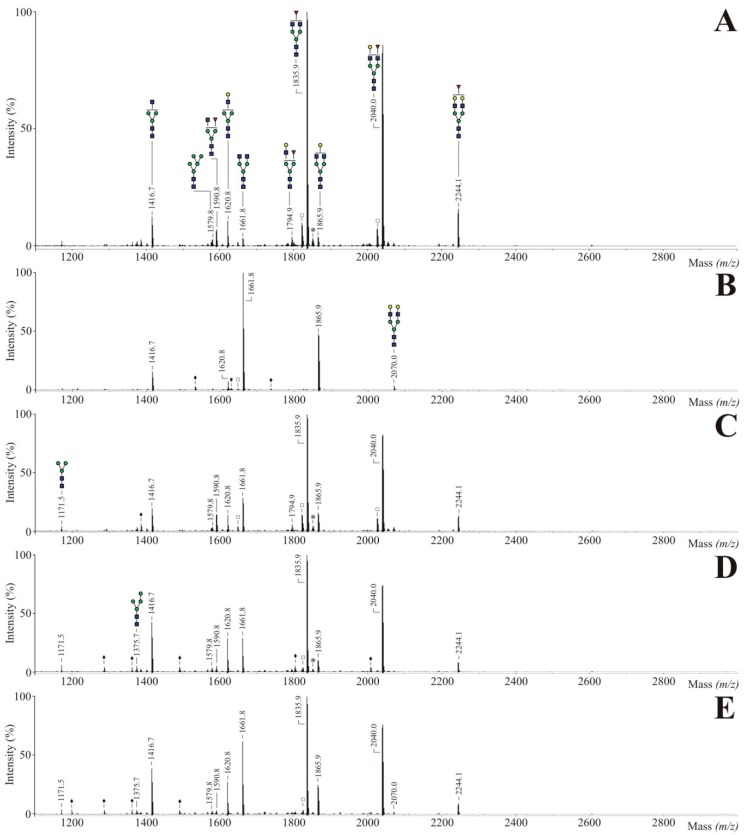
MALDI-TOF mass spectra of permethylated *N-*glycans of purified trastuzumab after three-day treatment of CHO RMD cells with 80 µM of Ac_4_Fuc (**C**), Prop_4_Fuc (**D**) or PEG_4_Fuc (**E**) under serum-free culture conditions; in comparison to an untreated control of the CHO (**A**) or CHO RMD cell line (**B**). *N*-linked glycans were released enzymatically using *N*-glycosidase F. Molecular ions are present in either sodiated [M + Na]^+^ or potassiated [M + K]^+^ form (*). Permethylation artifacts are marked by an unfilled square (□) and unidentified peaks by a black diamond (♦). All signals with relative peak areas higher than 0.5% are labeled with their respective numerical *m/z*-values. Graphical visualization of all structures is only done once per *m/z* in the whole Figure (**A**–**E**).

Furthermore, we compared the trastuzumab *N*-glycosylation profiles obtained from CHO RMD cells depending on the application of the different fucose analogues. The CHO RMD cell line was supplied with 80 µM of Ac_4_Fuc, Prop_4_Fuc or PEG_4_Fuc, while untreated references of CHO RMD and CHO cells were cultivated in parallel. Subsequent to recombinant protein production, all cell supernatants were subjected to protein G affinity purification. Respectively, 40 µg of purified trastuzumab underwent tryptic cleavage into glycopeptides, enzymatic release of *N-*linked glycans by *N-*glycosidase F and permethylation. The composition of IgG *N-*glycan structures is shown in the MALDI-TOF spectra covering a mass range of *m/z* 1000–3000 (Figure 5A–E). As reported previously for desialylated trastuzumab *N‑*glycans of untreated CHO cells [22], monofucosylated biantennary structures bearing none, one or two galactose residues (Figure 5A, *m/z* 1835.9, 2040.0 or 2244.1) were the most abundant structures in our study as well. Thus, the functional fucosylation pathway was demonstrated additionally on the structural level of the CHO IgG *N*-glycans. In contrast, the mass spectrum of CHO RMD *N-*glycans of trastuzumab (Figure 5B) revealed no fucosylated structures at all and showed, hence, afucosylated biantennary molecules with varying galactosylation (*m/z* 1661.8, 1865.9 and 2070.0) as prominent signals. Generally, antibodies expressed in CHO cells differ from native human IgGs: they lack the bisecting GlcNAc branch (due to the absence of the relevant transferase enzyme activity [35]) and often show a higher fraction of highmannose-type *N-*glycans (see *m/z* 1375.7 and 1579.6) that is, above all, characteristic for recombinant antibody expression as stated in [40]. Treating CHO RMD cells with 80 µM of acylated fucoses (Figure 5C–E) could qualitatively compensate their fucosylation defect and resulted in mass spectra similar to the CHO reference cell line (see Figure 5A). According to the relative portions of a few afucosylated structures (*m/z* 1661.8 and 1865.9) and fucosylated peaks (*m/z* 1590.8 or 2244.1), the approach of Ac_4_Fuc (C) resulted in a slightly higher antibody fucosylation than Prop_4_Fuc (D) or PEG_4_Fuc (E) under equal conditions.

**Figure 6:**
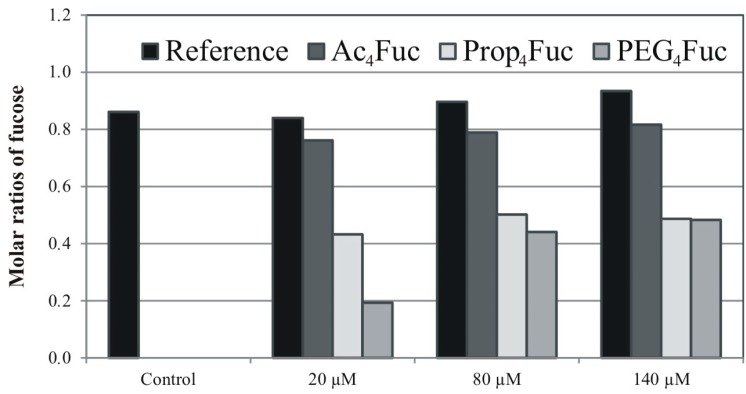
Molar ratios of fucose derived from glycans of recombinant trastuzumab as determined by HPAEC-PAD. CHO RMD cells were treated with varying concentrations of Ac_4_Fuc, Prop_4_Fuc and PEG_4_Fuc (compared to an untreated control) under serum-free conditions for three days. Treatment of parental CHO cells was performed likewise and is exemplarily shown for Ac_4_Fuc (termed as “Reference”). Ratios are given related to mannose = 3 (based on the composition of a *N*-glycan core structure).

In order to measure these effects on the platform of recombinant trastuzumab, we subsequently performed monosaccharide analysis by HPAEC-PAD. Since the effective concentrations of each analogue may also differ and, accordingly, be lower or higher than 80 µM, the experimental set up was expanded to the concentrations of 20 µM and 140 µM. We also aimed to investigate if the application of fucose analogues to CHO RMD cells may quantitatively reach the trastuzumab fucosylation level of the CHO reference cell line, and, if this level can be further increased by metabolic glycoengineering. For all approaches, 20 µg of purified IgG therefore underwent strong acid hydrolysis prior to monosaccharide separation by anion exchange chromatography. Resulting data of molar fucose ratios are summarized in Figure 6.

Monosaccharide analysis of trastuzumab *N-*glycans confirmed the results of MALDI-TOF mass spectrometry on a quantitative level. Irrespective of the applied concentrations, treatment of CHO RMD cells with Ac_4_Fuc achieved the highest degree of trastuzumab fucosylation. Ac_4_Fuc feeding of CHO RMD cells resulted in about 90% fucosylation compared to trastuzumab from untreated CHO reference cells. This acylated fucose variant showed the most effective utilization for glycan synthesis at a concentration of 140 µM, but concentrations of 20 µM and 80 µM resulted in similar fucose ratios. Interestingly, PEG_4_Fuc was incorporated far less into trastuzumab glycans, in particular when applied at low concentrations. In comparison to the reference of untreated CHO cells, only about 20% (at 20 µM) or 50% (at 80 µM) of trastuzumab fucosylation was obtained for PEG_4_Fuc treatment. One potential reason may be that the compound termed as PEG_4_Fuc within this study was, in fact, a mixture of PEG_3_Fuc and PEG_4_Fuc (see Syntheses of Fucose Analogues, Supplementary Information). Due to the missing of one acyl group, the membrane permeability of PEG_3_Fuc might be slightly reduced compared to PEG_4_Fuc and, in consequence, affecting the metabolic outcome and intracellular efficiency of this compound mixture. However, the amount of PEG_3_Fuc in the mixture was maximal 50%, and the application of the fourfold concentration (80 µM instead of 20 µM) of the mixture did result in a much lower incorporation rate, compared to the effects of 20 µM Ac_4_Fuc. A probable cause rather seems to be the PEG modification itself. Due to its size, the diffusion across the plasma membrane can be decelerated. Moreover, the activity of the unspecific cytosolic esterases cleaving the acyl moieties might be affected. Although it is reported for Prop_4_ManNAc that these enzymes are also active on longer ester derivatives [21], the fucose PEGylation might be removed less effective.

Regarding trastuzumab produced in the CHO reference cell line, only a minor increase in fucosylation resulted when these cells were treated with raising concentrations of Ac_4_Fuc—although this was the most effective analogue in CHO RMD approaches. Thus, CHO wild type cells with functional glycosylation machinery already seem to utilize their entire fucosylation capacity. Their application for metabolic glycoengineering approaches is restricted due to the limits of the fucose biosynthesis pathway, in particular the activity of fucosyltransferases. These findings are in contrast to results of Gu and Wang [16], who further increased the sialylation level of human interferon-γ by metabolic glycoengineering in CHO cells with a functional sialic acid pathway.

### 3.4. Effects of Sequential Trastuzumab Fucosylation on FcγRIIIa Binding

**Figure 7:**
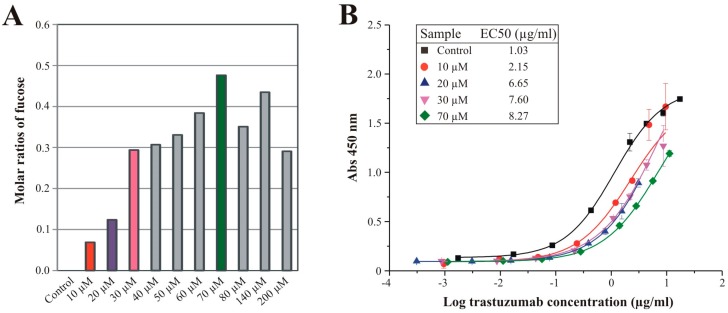
(**A**): Molar ratios of fucose derived from glycans of recombinant trastuzumab as determined by HPAEC-PAD. CHO RMD cells were treated with raising concentrations of PEG_4_Fuc (compared to an untreated control) under serum-free conditions for three days. Ratios are given related to mannose = 3 (based on the composition of a *N*-glycan core structure); (**B**): Binding curves of histidine-tagged FcγRIIIa to trastuzumab variants exhibiting differential fucosylation in consequence of PEG_4_Fuc treatment of the CHO RMD expression cell line. Cells were supplied with selected concentrations of PEGylated fucose (compared to an untreated control) under serum-free conditions for three days. Respectively, purified trastuzumab samples were analyzed in a FcγRIIIa binding assay. Points indicate the median absorption at 450 nm ± deviations of duplicates. The inset table displays the median EC50 values determined by a four-parameter logistic curve fit model.

Since the HPAEC-PAD monosaccharide analysis of trastuzumab glycans (Figure 6) revealed a stepwise increasing fucose amount when PEG_4_Fuc was fed to CHO RMD cells, we aimed at a sequential fucosylation of the recombinant antibody based on PEG_4_Fuc treatment in concentration stages between 0 and 200 µM. As stated above, treated CHO RMD cells were incubated again for three days in terms of recombinant protein production followed by affinity purification of trastuzumab. Effects on recombinant protein level were determined first by lectin blot analysis (Figure S5) that showed, in particular for concentrations of 10–50 µM, a stepwise enhancement of fucose-specific AAL-I signal intensity for the antibody’s heavy chains. Quantitative analysis by HPAEC-PAD visualizes the molar ratios of fucose in Figure 7A. Up to a concentration of 70 µM PEG_4_Fuc, the fucose amount of trastuzumab glycans was increased stepwise while concentrations higher than 70 µM could not raise the fucosylation status furthermore.

In order to demonstrate the consequences of defined fucosylation levels on the antibody’s receptor binding activity, we performed an FcγRIIIa binding assay for selected samples with an increasing degree of fucosylation. Figure 7B shows the concentration-dependent absorption of purified CHO RMD/trastuzumab samples. Confirming previously published work, binding curves and resulting median EC50 values of the trastuzumab samples indicate decreased binding to FcγRIIIa for fucosylated antibody variants compared to the afucosylated control. Furthermore, we could show correlation between the raising fucosylation level and reduction of trastuzumab binding activity to FcγRIIIa.

Analyzing the binding of purified trastuzumab to the ADCC-relevant receptor allows predicting ADCC activity of IgG antibody samples [22]. Accordingly, we demonstrate by means of the acylated fucose analogue PEG_4_Fuc applied for CHO RMD cells, that metabolic glycoengineering is an appropriate tool to directly influence glycosylation and, thus, functional aspects of a recombinant model glycoprotein.

## 4. Conclusions

We reported the first characterization of novel fucose analogues for metabolic glycoengineering of recombinant glycoproteins as a proof-of-principle. Our data reveal that the fucose analogues are of low toxicity for cells used for glycoprotein production in concentrations suitable for optimal glycoengineering. This allows their application also in larger production scales. Further investigations have to clarify, if a controlled bioprocess would allow lower concentrations of fucose analogues for reduced costs, and maintenance of the producing cell lines for a longer period than the three days of incubation in this study.

We further demonstrated, that metabolic glycoengineering by the use of a cell system with a blocked *de novo* pathway in combination with particular fucose analogues (in this case PEG_4_Fuc) results in gradual, functionally relevant fucosylation of a recombinant glycoprotein. Although in this study the increase of fucosylation of a therapeutic antibody results in decrease of its desired function, the technique developed by us should be worth to be applied to other fucosylated glycoproteins important for the numerous fucose-dependent cellular functions [6]. In addition, other important mono-saccharides of glycans could be targets by the use of appropriate analogues. In this context, a gradual sialylation could be of particular interest, due to the important function of this sugar in the efficacy of recombinant glycoproteins [42,43]. Cell lines with defects in the *de novo* sialic acid synthesis [11,44] could be addressed by the application of acylated ManNAc or sialic acid analogues.

## Supplementary Files

Supplementary File 1
