# Supplementary material for: Applying Acylated Fucose Analogues to Metabolic Glycoengineering
Source: Bioengineering (Basel). 2015 Nov 30;2(4):213–34. doi: 10.3390/bioengineering2040213 (PMC5597091; doi:10.3390/bioengineering2040213)
Supplement: Supplementary File 1 [file bioengineering-02-00213-s001.pdf]

Applying Acylated Fucose Analogues to Metabolic Glycoengineering

Syntheses of Fucose Analogues

Acetylated Fucose (Ac_4Fuc)

According to a procedure of Duléry *et al.* (Carbohydrate Research 342, 2007, pp. 894–900) 0.51 g (3.09 mmol) L-fucose were stirred in a mixture of acetic anhydride and pyridine 1:1 (20 mL) overnight. The solvent was evaporated as an azeotrope with toluene to dryness. 1.07 g of the crude product were obtained. A fraction was purified via column chromatography (cyclohexane/ethyl acetate 2:1) to yield 534 mg (52%) of the clean peracetylated fucose. Spectroscopic data were consistent with those previously reported.

Propanoylated Fucose ($Prop_4Fuc$)

This fucose analogue was prepared in analogy to the peracetylation procedure described above. 0.49 g (2.98 mmol) L-fucose were stirred in propionic anhydride/pyridine (20 mL, 1:1) overnight. The solvent was removed *in vacuo* and the residual pyridine was evaporated as an azeotrope with toluene. 1.28 g of the crude product were synthesized and purified by column chromatography (cyclohexane/ethyl acetate 8:1 to 6:1). 0.99 g (98%) of the pure product were obtained as an anomeric mixture.

^{1}H-NMR (300 MHz, $CDCl_3$): major (α-) anomer: δ = 6.37 (d, ^3J = 2.1 Hz, 1H), 5.40–5.35 (m, 1H), 5.38 (s, 2H), 4.29 (q, ^3J = 6.5 Hz, 1H), 2.45 (dq, ^2J = 12.6 Hz, ^3J = 7.6 Hz, 4H), 2.26 (qd, J = 7.6 Hz, 4.1 Hz, 4H), 1.30–1.04 (m, 15H); minor (β-) anomer: δ = 5.70 (d, J = 8.3 Hz, 1H), 5.33–5.17 (m, 2H), 5.15–5.03 (m, 1H), 3.96 (q, J = 6.2 Hz, 1H), 2.52–2.11 (m, 8H), 1.24–0.97 (m, 15H).

^{13}C-NMR (75 MHz, $CDCl_3$): major (α-) anomer: δ = 174.00 (C=O), 173.48 (C=O), 173.30 (C=O), 172.60 (C=O), 89.84 (O-C-O), 70.39 (CH), 67.82 (CH), 67.31 (CH), 66.41 (CH), 27.54 (CH_2), 27.40 (CH_2), 27.35 (CH_2), 27.25 (CH_2), 15.94 (CH_3), 9.34 (CH_2), 9.00 (CH_3), 8.89 (CH_3), 8.83 (CH_3); minor (β-) anomer: δ = 173.98 (C=O), 173.55 (C=O), 172.93 (C=O), 172.76 (C=O), 92.06 (O-C-O), 71.13 (CH), 70.31 (CH), 69.77 (CH), 67.79 (CH), 27.38 (CH_2), 27.30 (CH_2), 27.27 (CH_2), 27.12 (CH_2), 15.90 (CH_3), 9.16 (CH_3), 8.78 (CH_3), 8.75 (CH_3), 8.68 (CH_3).

HR-MS (ESI-TOF): $[M + Na]^+$: 411.1659, calc.: 411.1626.

IR (cm^{-1}): 806 *w*, 892 *w*, 1039 *m* (C-O), 1171 *s* (C-O), 1274 *w*, 1356 *w*, 1463 w, 1767 *vs.* (C=O), 2343 *m*, 2359 *m*, 2986 *w* (C-H).

PEGylated Fucose (PEG_4Fuc), Mixture of Compounds

The 2-[2-(2-methoxyethoxy)ethoxy] acetyl chloride was prepared according to a procedure of Lafitte *et al.* (The Journal of Organic Chemistry, 2005, pp. 2701–2707). 2.8 mL (2.41 g, 13.5 mmol) 2-[2-(2-methoxyethoxy)ethoxy] acetic acid were dissolved in 60 mL dry dichloromethane. 2.8 mL (1.72 g, 14.4 mmol) thionyl chloride were added dropwise at room temperature. The reaction mixture was

refluxed overnight. The solvent was removed *in vacuo* and the residue was dissolved in 6 mL of dry acetone. 3 mL of the solution were added to the suspension of L-fucose (0.52 g, 3.2 mmol) in acetone in the presence of 1.82 mL (2.6 g, 25 mmol) triethylamine. The reaction mixture was stirred overnight and filtered. The solvent was removed and the residue was taken up in dichloromethane. The excess of 2-[2-(2-methoxyethoxy)ethoxy] acetic acid was removed via dialysis using benzoylated cellulose tubing (MWCO 500 Da, Sigma-Aldrich GmbH, Taufkirchen, Germany). Subsequent to evaporation of the solvent, 320 mg (14%) of the product were obtained. The conversion was incomplete with respect to the acetal position. Evaluation of the PEG_3Fuc/PEG_4Fuc mixture by ^{1}H-NMR data (see below) and HR-MS analysis revealed a portion of PEG_3Fuc of maximal 50%. This mixture was used for all experiments within this work.

^{1}H-NMR (400 MHz, $CDCl_3$): δ = 6.41–6.38 (m, 0.66H, α), 5.78–5.79 (m, 0.33H, β), 5.60–5.10 (m, 3H), 4.35–3.2 (m, (55 + 42)/2H), 1.4–1.1 H (m, 3H).

^{13}C-NMR (75 MHz, $CDCl_3$): δ = 170.40 (C=O), 170.20 (C=O), 170.07 (C=O), 169.73 (C=O), 169.65 (C=O), 169.48 (C=O), 169.48 (C=O), 169.42 (C=O), 169.37 (C=O), 169.08 (C=O), 168.88 (C=O), 168.88 (C=O), 168.76 (C=O), 168.62 (C=O), 93.27 (O-C-O), 92.06 (O-C-O), 90.07 (O-C-O), 82.41 (O-C-O), 76.58 (CH), 76.13 (CH), 75.58 (CH), 74.1 (CH), 73.93 (CH), 71.71 (O-CH_3), 71.58 (O-CH_3), 71.0–70.0 (m, CH_2), 69.0–68.6 (m, CH), 68.4–67.8 (m, CH_2), 67.34 (CH), 66.49 (CH), 58.88 (CH_2), 58.86 (CH_2), 58.81 (CH_2), 53.36 (CH_2), 16.05 (CH_3), 15.88 (CH_3), 15.76 (CH_3), 15.60 (CH_3), 8.46 (CH_3).

HR-MS (ESI-TOF): $[M + Na]^+$: 827.3519, calc.: 827.3543; $[M(PEG_3) + Na]^+$: 667.2783, calc.: 667.2704.

IR (cm^{-1}): 851 *m*, 930 *m*, 965 *m*, 1031 *s*, 1103 *vs.* (C-O), 1197 *s*, 1247 *m*, 1364 *w*, 1455 *w*, 1759 *s* (C=O), 2878 *m* (C-H).

NMR Spectra

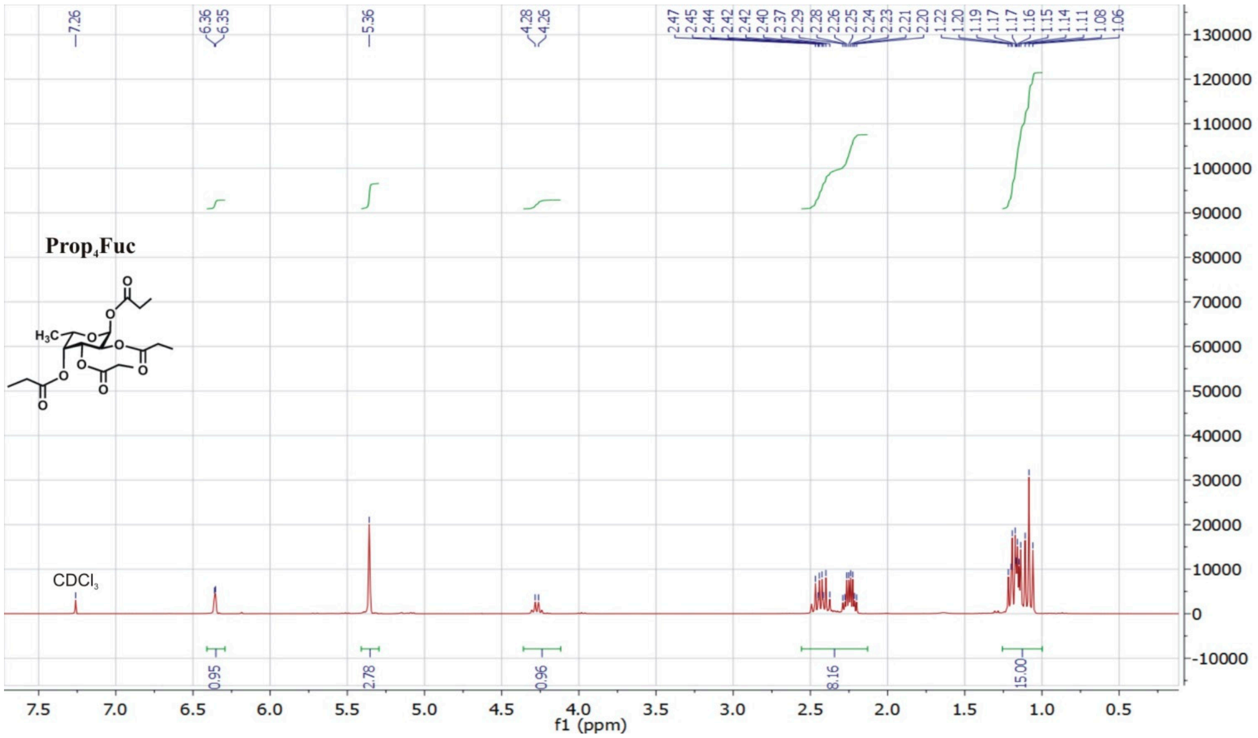

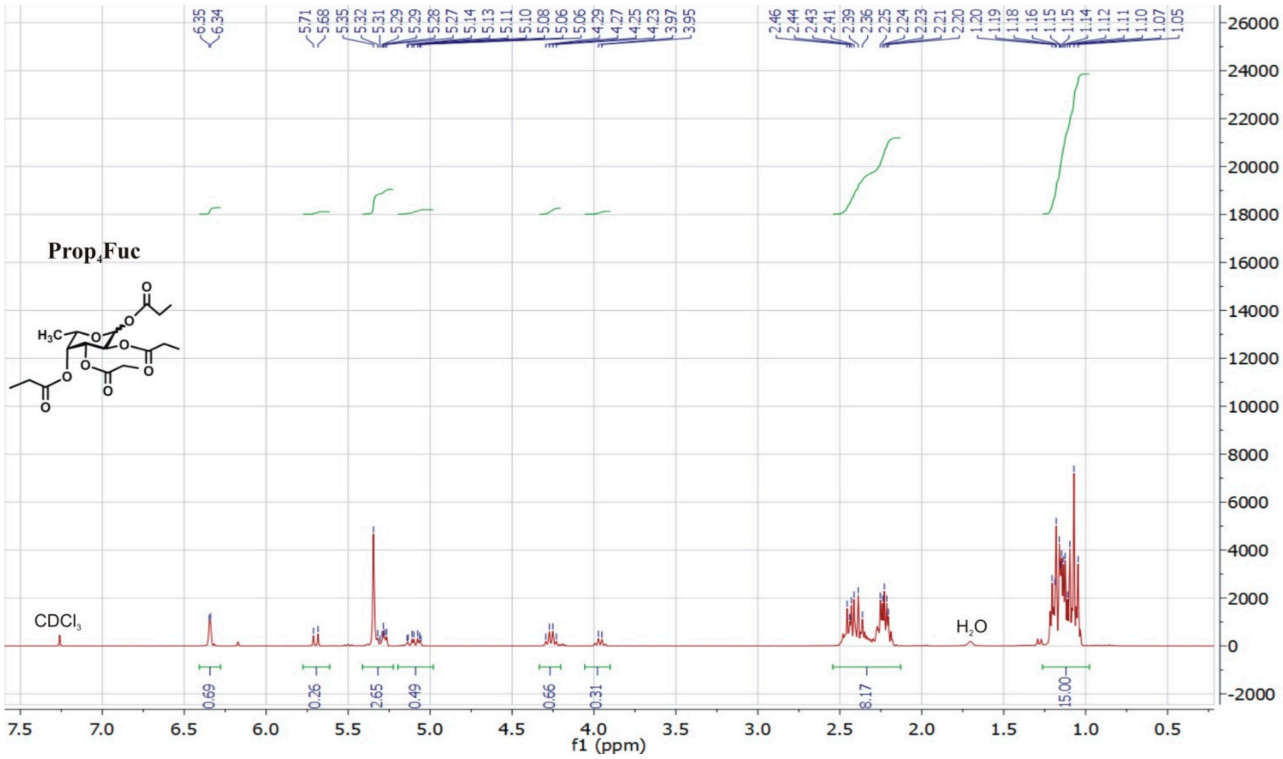
Prop4Fuc
CDCl3
H2O
f1 (ppm)

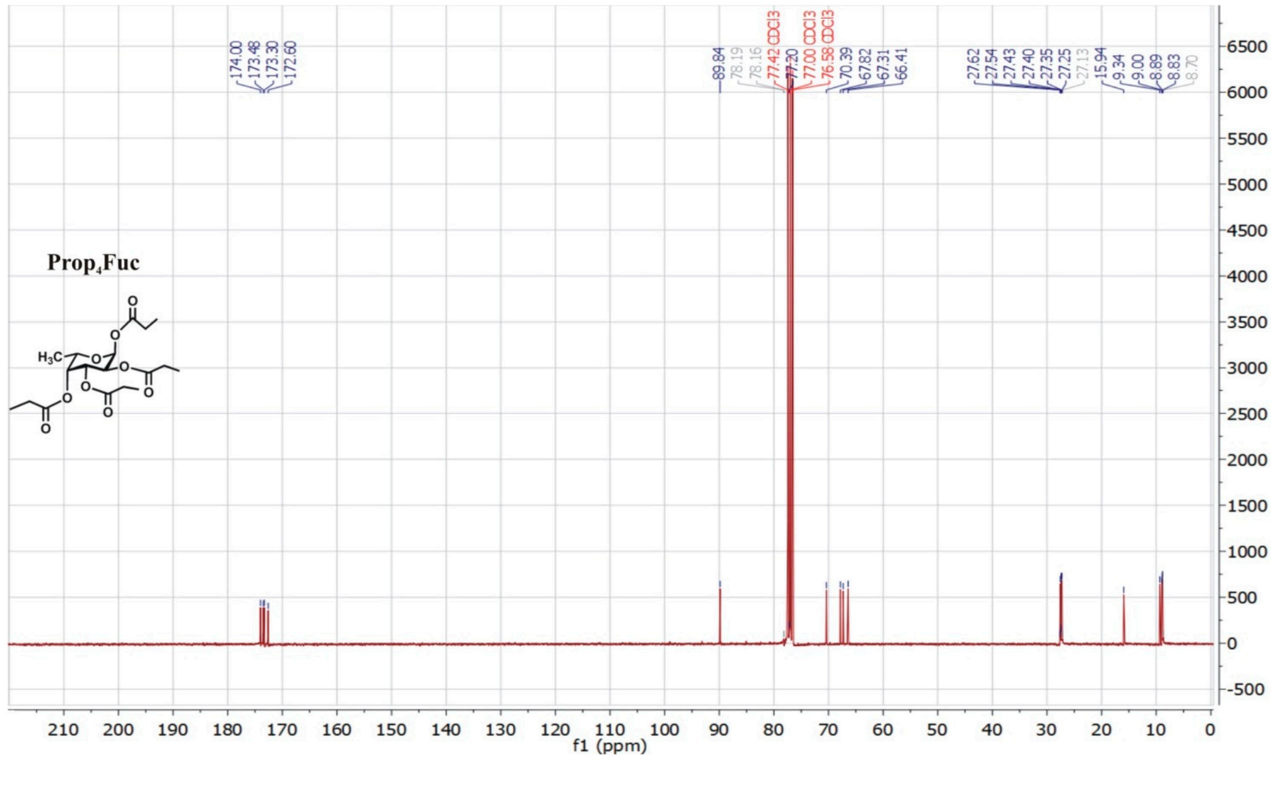
Prop4Fuc
f1 (ppm)

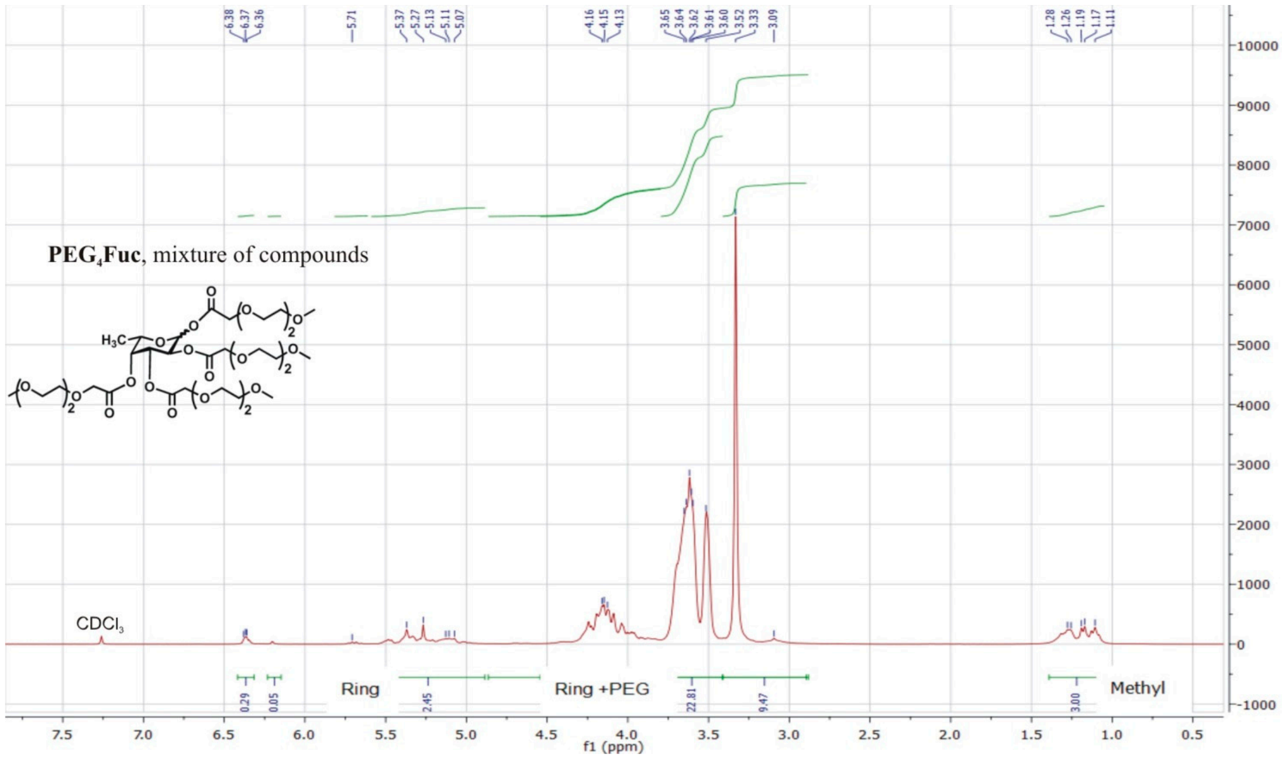
PEG$_4$Fuc, mixture of compounds
CDCl$_3$
Ring
Ring +PEG
Methyl
f1 (ppm)

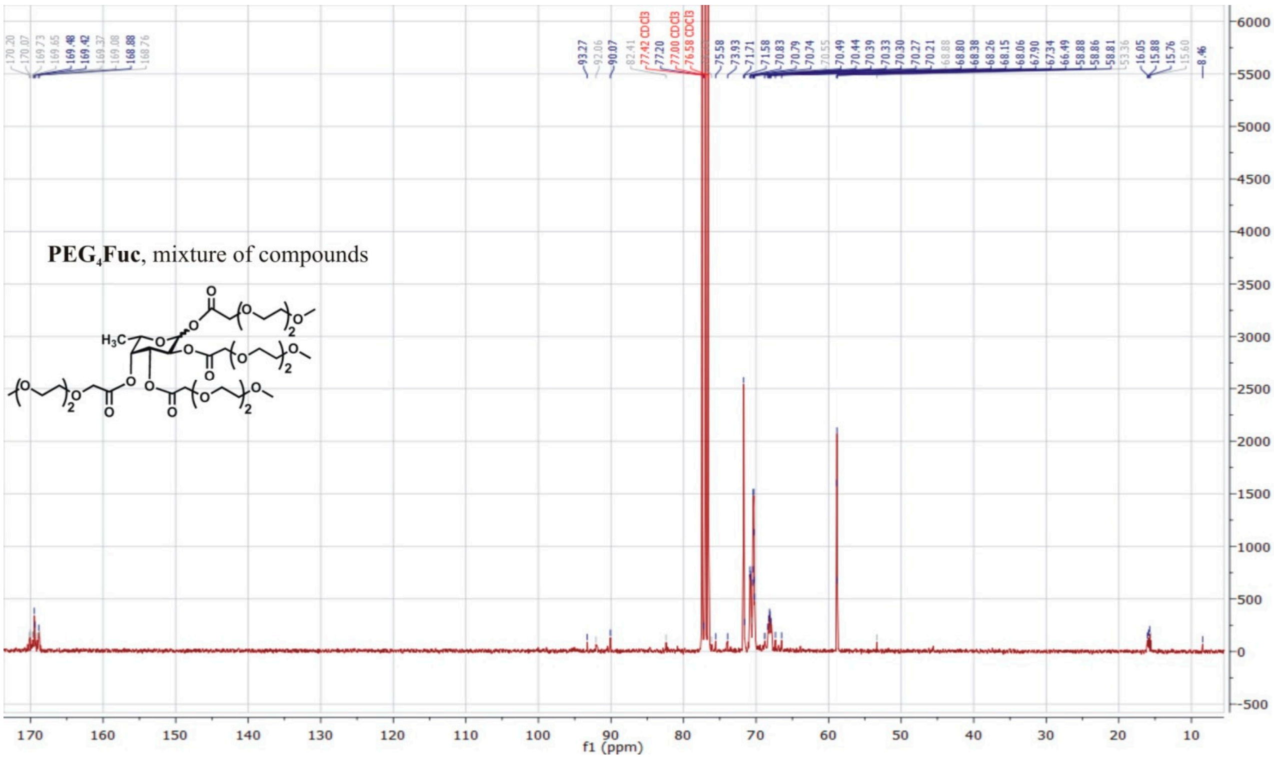
PEG$_4$Fuc, mixture of compounds
f1 (ppm)

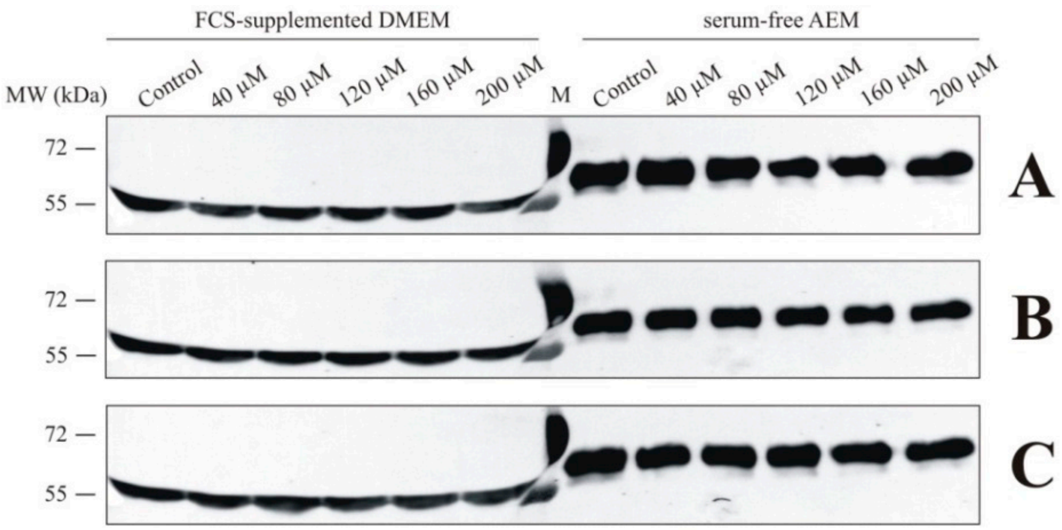

Figure S1. Western blot analyses of recombinant A1AT expressed in HEK-293T cells subsequent to a treatment with varying concentrations of Ac_4Fuc (**A**); $Prop_4Fuc$ (**B**); or PEG_4Fuc (**C**) (compared to an untreated control) for three days. Serum-containing and serum-free cell supernatants were separated via reducing SDS-PAGE and electroblotted prior to immunologic detection by a peroxidase-coupled antibody to human A1AT. The altered appearance of the A1AT protein band in the presence and absence of FCS can be referred to the interference of BSA (66 kDa) which affects the migration of A1AT.

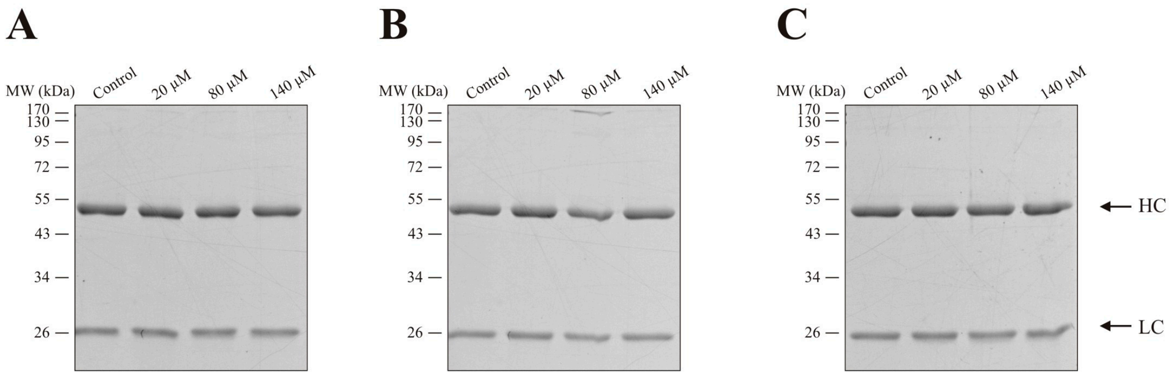

Figure S2. Recombinant production of trastuzumab in CHO RMD cells subsequent to a treatment with varying concentrations of Ac_4Fuc (**A**); $Prop_4Fuc$ (**B**); and PEG_4Fuc (**C**) (compared to an untreated control) for three days under serum-free conditions. Supernatants (that contain the recombinant antibody) were simultaneously purified using protein G resin and identical sample volumes were subjected to reducing SDS-PAGE and Coomassie staining. Heavy (HC) and light chain (LC) are marked with arrows.

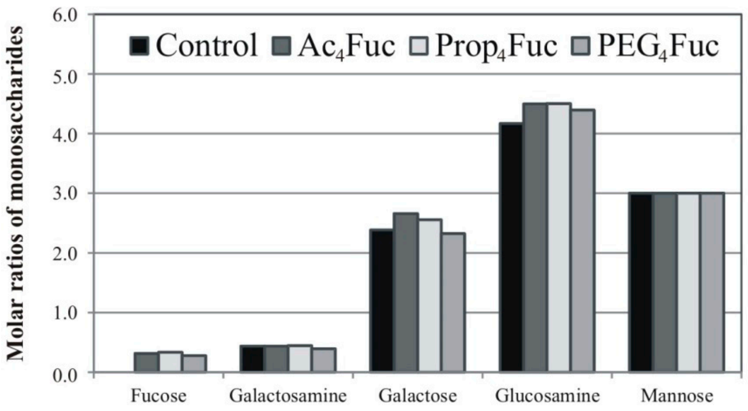

Figure S3. Molar ratios of monosaccharides derived from glycans of CHO RMD membrane proteins as determined by HPAEC-PAD. CHO RMD cells were treated with 80 µM of the respective fucose analogue (compared to an untreated control) under serum-free conditions for three days. Ratios are given related to mannose = 3 (based on the composition of a *N*-glycan core structure). Caused by the conditions of strong acid hydrolysis, sialic acids are not detectable while GalNAc and GlcNAc were measured as galactosamine and glucosamine, respectively.

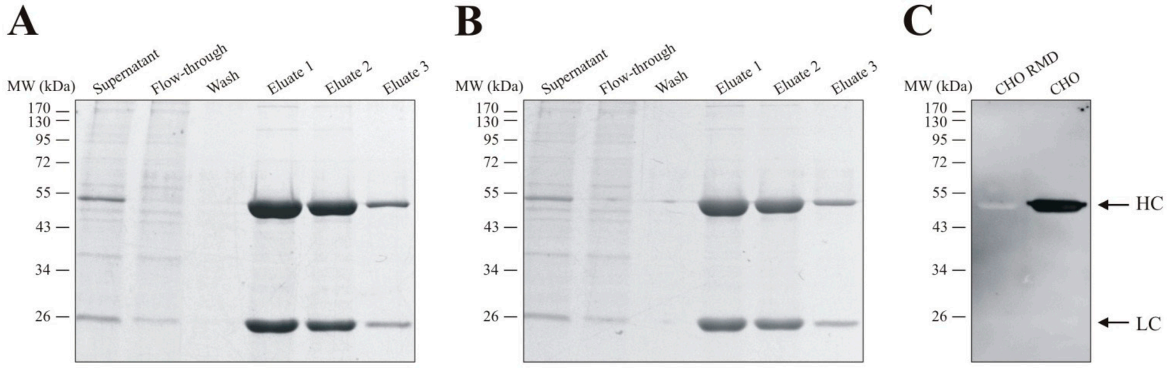

Figure S4. Affinity purification using protein G resin (**A**, **B**) and lectin blot analysis (**C**) of recombinant trastuzumab expressed in CHO cells under serum-free conditions. Sample purity and integrity is documented for expression in CHO RMD (**A**) and CHO cells (**B**) subsequent to separation via reducing SDS-PAGE and Coomassie staining. Respectively, 1 µg of the purified antibody was separated likewise, electroblotted and probed with AAL-I for lectin staining shown in (**C**). Heavy (HC) and light chain (LC) are marked with arrows.

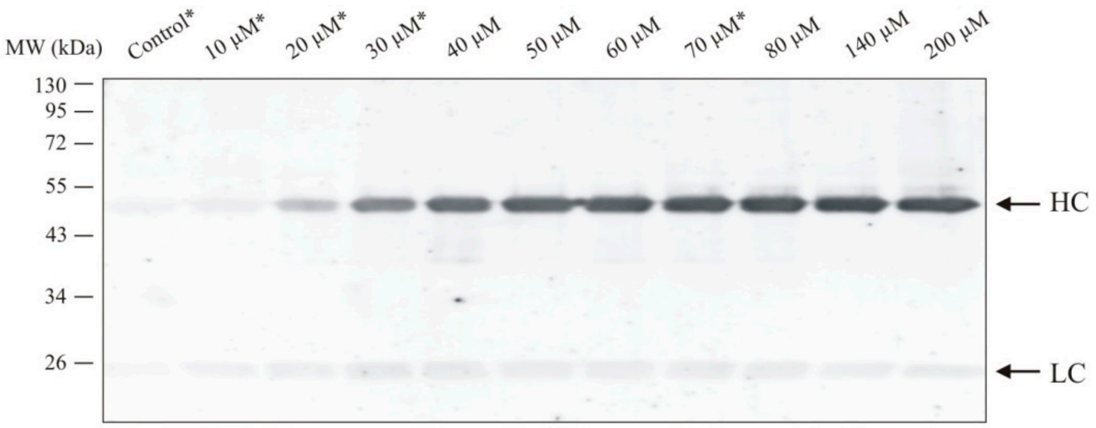

Figure S5. Lectin blot analysis of recombinant trastuzumab expressed in CHO RMD cells. Cell treatment with raising concentrations of PEG_4Fuc for three days (compared to an untreated control) under serum-free conditions was followed by affinity purification of trastuzumab. Respectively, 0.8 µg of the purified antibody was separated by reducing SDS-PAGE, electroblotted and probed with AAL-I. Heavy (HC) and light chain (LC) are marked with arrows. Asterisks (*) indicate samples that were subsequently referred to FcγRIIIa binding assay.

© 2015 by the authors; licensee MDPI, Basel, Switzerland. This article is an open access article distributed under the terms and conditions of the Creative Commons Attribution license (http://creativecommons.org/licenses/by/4.0/).
